# Quantifying the extent of human disturbance activities and anthropogenic stressors in wetlands across the conterminous United States: results from the National Wetland Condition Assessment

**DOI:** 10.1007/s10661-019-7314-6

**Published:** 2019-06-20

**Authors:** Gregg A. Lomnicky, Alan T. Herlihy, Philip R. Kaufmann

**Affiliations:** 1 CSS Inc., 200 SW 35th St, Corvallis, OR 97333 USA; 2 Department of Fisheries and Wildlife, Oregon State University, 104 Nash Hall, Corvallis, OR 97331 USA; 3 Office of Research and Development, National Health and Environmental Effects Research Laboratory- Western Ecology Division, US Environmental Protection Agency, 200 SW 35th St, Corvallis, OR 97333 USA

**Keywords:** Wetlands, Human disturbance, Indicators, Stressor, Buffer, Hydrology

## Abstract

In 2011, the U.S. Environmental Protection Agency conducted the National Wetland Condition Assessment (NWCA) as part of the National Aquatic Resource Survey (NARS) program to determine the condition of wetlands across the 48 contiguous states of the United States (US). Sites were selected using a generalized random tessellated stratified (GRTS) probability design. We quantified the types, extent, and magnitude of human activities as indicators of potential stress on a sample of 1138 wetland sites representing a target population of 251,546 km^2^ of wetlands in the US. We used field observations of the presence and proximity of more than 50 pre-determined types of human activity to define two types of indices that quantify human influences on wetlands. We grouped these observations into five types of human activity (classes) and summed them within and across these classes to define five metrics and an overall Human Disturbance Activity Index (HDAI). We calculated six Anthropogenic Stress Indices (ASIs) by summing human disturbance activity observations within stressor categories according to their expected effect on each of six aspects of wetland condition. Based on repeat-visit data, the precision of these metrics and indices was sufficient for regional and national assessments. Among the six categories of stress assessed nationally, the percentage of wetland area having ASI levels indicating high stress levels ranged from 10% due to filling/erosional activities to 27% due to vegetation removal activities. The proportion of wetland area with no signs of human disturbance activity (HDAI = 0) within a 140-m diameter area varied widely among the different wetland ecoregions/types we assessed. No visible human disturbance activity was evident in 70% of estuarine wetlands, but among non-estuarine wetlands, only 8% of the wetland area in the West, 15% of the Interior Plains, 22% of the Coastal Plains, and 36% of the Eastern Mountains and Upper Midwest lacked visible evidence of disturbance. The woody wetlands of the West were the most highly stressed reporting group, with more than 75% of their wetland area subject to high levels of ditching, hardening, and vegetation removal. The NWCA offers a unique opportunity to quantify the type, intensity, and extent of human activities in and around wetlands and to assess their likely stress on wetland ecological functions, physical integrity, and overall condition at regional and continental scales.

## Introduction

Wetlands, which are distributed widely across the landscape in diverse settings, are an integral component of US aquatic resources. They occur as small isolated patches in mountain meadows, as strips along rivers and streams, and as vast complexes along the southern and eastern coasts of the US. Wetlands include swamps, marshes, and bogs, but also encompass edge habitat along lakes, streams, and rivers. Wherever they are present, wetlands function as natural sponges in the landscape, absorbing runoff and filtering surface water, thereby capturing excess sediment, nutrients, and other pollutants (Johnston 1991). To support natural ecological processes occurring in wetlands including hydrology, soil, and vegetation development, wetlands function best with a minimally disturbed surrounding area, just as riverine systems benefit from an intact riparian zone. Consequently, the presence, extent, and general condition of this “buffering” area surrounding the wetland influence the ecological condition of the wetland (Norman 1996).

Wetlands and their surrounding buffer areas provide many benefits. They offer essential wildlife habitat (Naugle et al. 2000; Brinson and Malvarez 2002), and natural vegetation surrounding and within wetlands provides food and habitat structure, benefiting both facultative and obligate wetland fauna. Wetlands are important for recreation and other ecosystem services for human benefit including hunting, food supply, and shelter (MEA 2005). Further, wetlands sequester carbon, control flooding, maintain biodiversity, foster fish and game bird production, and recharge aquifers (Keddy et al. 2009). Anthropogenic activities in and near wetlands have great potential to disturb wetland functions and degrade wetland condition, thereby diminishing the ecosystem services they provide.

Human disturbance of the landscape varies in distribution, intensity, and potential ecological impact by geographic region, ecosystem, and land use across the United States (US) (Niemi et al. 2004; USEPA 2016a, b). The natural functioning and processing of US wetland ecosystems are being compromised and degraded at multiple spatial and temporal scales across the landscape. For example, wetlands can be degraded in direct response to changes in local land use, or from more indirect influences such as airborne particulate fallout from distant urban centers. In the Western US, where water is scarce, levees, wells, diversion projects, and flood control alter the natural water regime of wetlands (Fretwell et al. 1996). Water management and land-drainage projects have greatly reduced wetland acreage for more than a century (Dahl et al. 1991), as landholders seek to retain water for extended seasonal use and/or convert wetlands to agriculture and urban use. Urban development has led to the infilling of wetlands to create suitable land for building (Dahl et al. 1991). Nationwide, many wetlands are under the continuing threat of resource extraction or draining to support development including building, farming, and human habitation as our population continues to grow. Identifying, measuring, and quantifying such human activities are critical to assessing the amount and degree to which humans are potentially affecting the physical and biological integrity of wetlands across the landscape.

The US Environmental Protection Agency’s (USEPA) National Aquatic Resource Surveys (NARS) have been implemented to generate statistically valid and policy-relevant reports on the condition of the nation’s aquatic resources. The National Wetland Condition Assessment (NWCA) is one of the NARS assessments, along with national surveys of lakes and reservoirs (USEPA 2016d), streams and rivers (USEPA 2016c), and estuaries (USEPA 2006). The first NWCA, conducted in 2011, was the first continental-scale wetland condition assessment across the conterminous United States. The goals of the NWCA are to (1) assess the ecological condition of wetlands and produce a national report describing the status of US wetlands and anthropogenic stressors commonly associated with poor condition; (2) collaborate with states and tribes in developing complementary monitoring tools, analytical approaches, and data management technology to aid wetland protection and restoration programs; and (3) advance the science of wetland monitoring and assessment to support wetland management needs.

In this paper, we use NWCA field observations of the presence and proximity of human activity, collected in ways similar to other NARS monitoring programs (Fennessy et al. 2007; USEPA 2016c, 2016d) to define two types of indices that quantify human influences on wetlands. We derived from the NWCA field forms a list of over 50 types of human activities that are likely to disrupt wetland ecological processes (primarily anthropogenic alterations of hydrology and vegetation). We then grouped these observations into five types of human activity (classes) and summed them within classes to define five metrics summarizing human activities in agriculture, residential and urban development, industry, hydrologic modification, and habitat modification. We also calculated an overall Human Disturbance Activity Index (HDAI) by summing those five human disturbance activity metrics. We further interpreted the human activity field observations to identify the likely level of anthropogenic stress on six separate aspects (categories) of wetland structure, function, and condition: vegetation replacement, vegetation removal, damming, ditching, hardening, and filling/erosion. Summing human disturbance activity observations within these six stressor categories, we derived six Anthropogenic Stress Indices (ASIs) that quantify the expected influence of human activity on each aspect. We used HDAI and ASI scores to assess the level of human activity and its expected stress on wetlands in the US. Specifically, the human activity classes (represented by five HDAI metrics) and stressor categories (represented by six ASIs) were used to define the extent of wetland area with levels of human activity or expected anthropogenic stress nationally, and by ecoregion and wetland type.

## Methods

### NWCA design

The NWCA was designed to assess the regional ecological condition of broad groups or populations of wetlands, rather than individual wetlands or wetlands within individual states or drainage basins. The NWCA design allows characterization of wetlands at national and regional scales using indicators of ecological condition and stress measured at individual wetland locations that comprise a statistically representative sample of a defined target population of wetlands. The NWCA target population for assessment includes all wetlands of the conterminous US not currently in crop production, tidal and non-tidal wetted areas with rooted vegetation, and, when open water is present, it is < 1 m deep (Olsen et al. 2019). A wetland’s jurisdictional status under state or federal regulatory programs did not factor into this target population definition.

Site selection was completed in two steps as described by Olsen et al. (2019). Because a consistent national digital map of all wetlands in the conterminous US was not available as a frame from which to draw the sample, the NWCA effort relied on the base map that is used for periodic reporting on the status and trends in wetlands area by the US Fish and Wildlife Service (USFWS) Wetland Status and Trends (S&T) program (Dahl 2011). Approximately 5000 4-mi^2^ plots based on 2005 aerial photography (the latest available from the USFWS S&T) were used to identify wetlands in the NWCA target population in the first site selection step. In the second step, a Generalized Random Tessellation Stratified (GRTS) survey design (Stevens and Olsen 1999, 2004) for an areal resource was applied to the S&T wetland polygons. This GRTS step was stratified by state and by NWCA wetland type with unequal probability of selection to ensure sufficient representation of less common wetland types, particularly in regions with less wetland area such as the western US (Olsen et al. 2012).

All potential sites from the NWCA survey design selection were screened before field visits using aerial photo interpretations and Geographic Information System (GIS) analyses to eliminate sites not suitable for NWCA sampling (e.g., wetland types not targeted by NWCA, wetlands converted to non-wetland land cover because of development). Sites were also eliminated from consideration during field reconnaissance if, for example, they were a non-target wetland type or could not be assessed because they were inaccessible or unsafe for crews to sample. Sites that were eliminated were systematically replaced from a randomized pool of replacement sites generated as part of the survey design. Details of the NWCA sampling design and site selection are fully described in the NWCA 2011 technical report (USEPA 2016a) and Olsen et al. (2019).

**Fig. 1:**
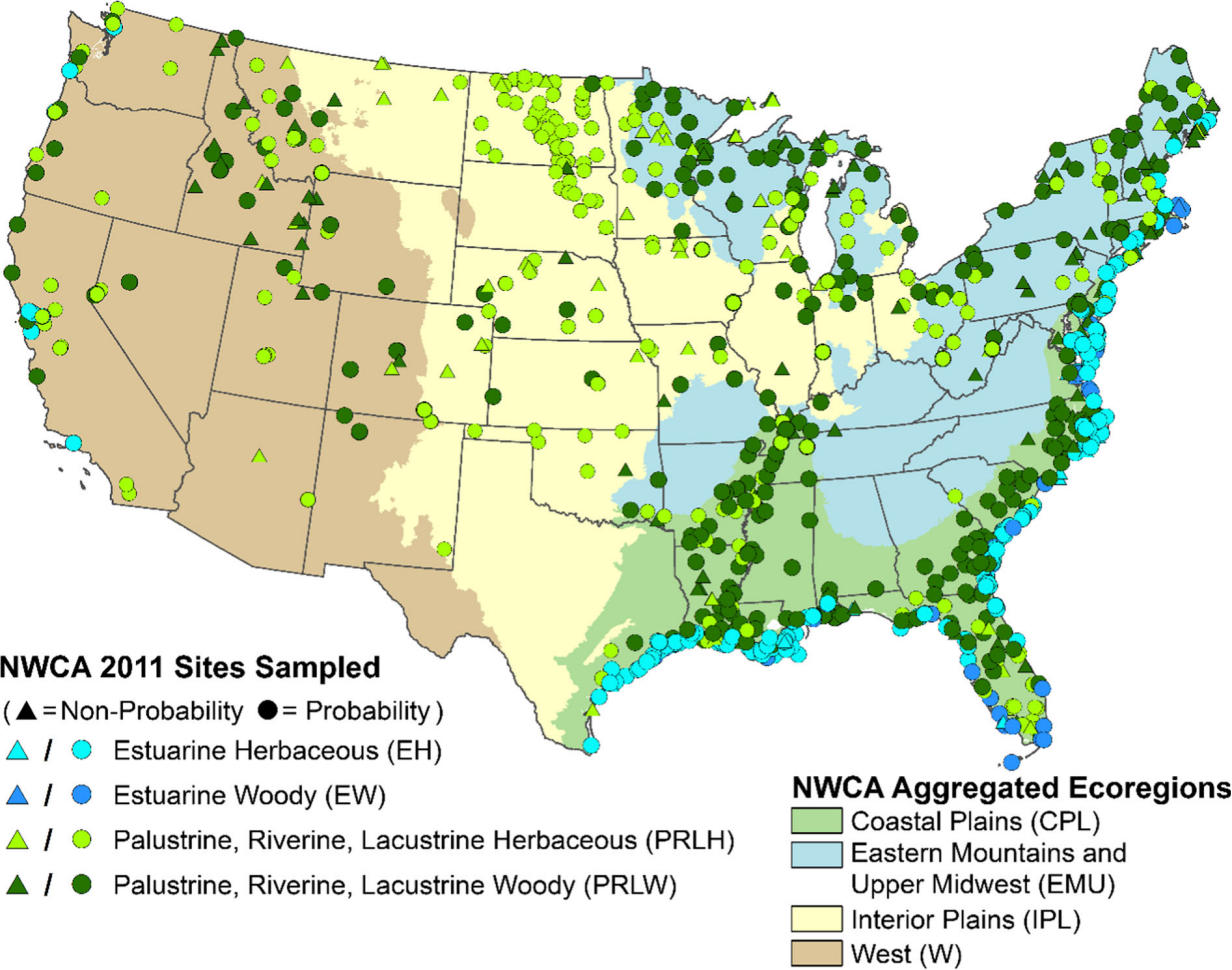
Location of 2011 NWCA sample sites by aggregated wetland type and within four aggregated ecoregions

A total of 1138 sites were sampled in the NWCA during 2011, of which 967 were probability sample sites (i.e., a statistical sample selection employing randomized systematic methods). Only the probability sites were used to make the national condition estimates (USEPA 2016a). The other 171 sites included 21 probability sites from a survey done by a state participating in the NWCA that did not meet the national design selection criteria for the NWCA and 150 sites that were handpicked with the intent to identify high-quality reference sites (Herlihy et al. 2019a). Sample sites were distributed throughout the conterminous US (Fig. 1). The spatial distribution of probability sites across the country was not uniform but followed the distribution of wetlands in the nation as represented in the original S&T (National Wetland Status and Trends) sample frame described by Dahl (2011).

A classification scheme for NWCA data analysis, incorporating both aggregated ecoregions and aggregated wetland types, was developed by Herlihy et al. (2019a) to account for continental-scale differences in wetland vegetation associated with regional differences in wetland chemistry, hydrology, ecology, and physical habitat (Table 1). The classification combined ecoregion and wetland type into 10 reporting groups. There were eight inland (non-estuarine) groups (the four aggregated ecoregions in Fig. 1, each with two aggregated wetland types—woody and herbaceous). Because the sample size was limited, we grouped all the US estuarine wetlands into only two groups (herbaceous and woody), each including wetlands from the Eastern, Gulf, and Western Coasts. The NWCA results on wetland ecological conditions and our results describing human activities and their likely associated anthropogenic stress are reported nationally (all sites) and by the 10 reporting groups. The NWCA probability sample sites represent a total target wetland area of 251,546 km^2^ (Table 1). Over 60% of the wetland area targeted by NWCA was found in just two of the reporting groups: woody palustrine, riverine, and lacustrine wetlands located in the Coastal Plain and the Eastern Mountains and Upper Midwest (CPL-PRLW and EMU-PRLW, Table 1).

**Table 1:** The 10 reporting groups in the NWCA and their sample size and estimated target wetland area

Reporting group code	Reporting group	Number of sites (all / probability)	Estimated target NWCA wetland area (km^2^ (%))
ALL-EH	All US estuarine-herbaceous	272 / 163	20,186 (8%)
ALL-EW	All US estuarine-woody	73 / 69	2015 (1%)
CPL-PRLH	Coastal Plains-palustrine, riverine, lacustrine herbaceous	72 / 62	15,178 (6%)
CPL-PRLW	Coastal Plains-palustrine, riverine, lacustrine woody	189 / 163	88,464 (35%)
EMU-PRLH	Eastern Mountains and Upper Midwest-palustrine, riverine, lacustrine herbaceous	73 / 55	15,225 (6%)
EMU-PRLW	Eastern Mountains and Upper Midwest-palustrine, riverine, lacustrine woody	127 / 83	65,421 (26%)
IPL-PRLH	Interior Plains-palustrine, riverine, lacustrine herbaceous	138 / 115	18,611 (7%)
IPL-PRLW	Interior Plains-palustrine, riverine, lacustrine woody	52 / 41	12,385 (5%)
W-PRLH	West-palustrine, riverine, lacustrine herbaceous	75 / 70	6023 (2%)
W-PRLW	West-palustrine, riverine, lacustrine woody	67 / 51	8037 (3%)
ALL	All U.S. (conterminous 48 States)	1138 / 967	251,546 (100%)

### NWCA field sampling

**Fig. 2:**
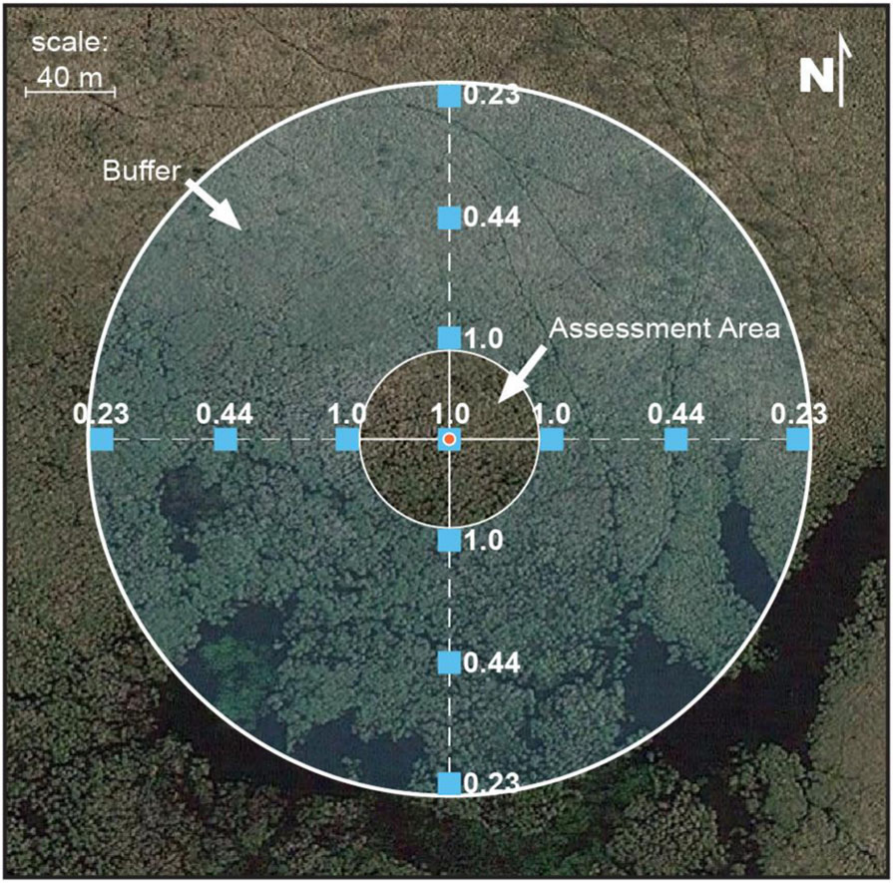
Field design layout for wetland sampling. A 40-m-radius (0.5 ha) assessment area (AA) was established at the designated survey design sampling location (red dot). The AA was nested within a buffer area extending 100 m away from the edge of the AA to delineate a plot with a 140-m radius (140-m plot). Hydrology-related human disturbance activities (only) were recorded throughout the AA. Human disturbance activities were tallied at 13 10 × 10 m square subplots indicated by blue squares within the 140-m plot. Proximity-weighting factors assigned to each subplot tally for calculating 140-m plot metric and index scores are shown next to each of the 13 subplots

Field methods for the NWCA are described in detail in the NWCA Field Operations Manual (USEPA 2011). Sampling was accomplished from April to September 2011 during an index period defined as the peak growing season when most vegetation is readily identified by their flowers or fruits. Additional rationale for sampling during this index period was to minimize seasonal variability and to accommodate logistical and safety considerations (USEPA 2016a). Centered on each sample location specified by the survey design, field crews established a 40-m-radius (0.5 ha) circular assessment area (AA) nested within a 140-m-radius plot (140-m plot) that included a buffer area extending an additional 100 m from the edge of the AA (Fig. 2). Vegetation and soil sampling were done within the AA, while evidence of human activities was assessed both within the AA and the 140-m plot.

Field crews observed and tallied the presence/absence of more than 50 pre-defined types of human activities (Table 2), as detailed in the NWCA *Field Operations Manual* (USEPA 2011). The human activity data came from two sources: (1) a checklist of 21 indicators of human activities likely to alter hydrology that were applied solely within the entire 40-m-radius AA (40-m plot), and (2) a more comprehensive checklist of over 50 indicators including human activities related to other wetland characteristics besides hydrologic alterations that was applied in subplots located within the 140-m plot (termed the 140-m plot tally). If crews noted an indicator of human activity that was not one of the pre-defined types on the field forms, they recorded and defined the activity in the “Other” category on the form. When calculating both the 40-m AA and the 140-m metrics, we assigned human activities in the Other category to one of the pre-defined types of human disturbance activity and stressor categories based on field crew comments.

**Table 2:** Field checklist of observations of human disturbance activity grouped for calculating five metrics of 140-m plot human disturbance activity

Human disturbance activity class	Field observations summed for the 140m-HDAI
Agriculture	Pasture/hay, range, row crops, fallow field, nursery, dairy, orchard, confined animal feeding operation, rural residential, gravel pit, irrigation
Residential and urban	Road (gravel, two-lane, or four-lane), parking lot/pavement, golf course, lawn/park, suburban residential, urban/multifamily, landfill, dumping, trash
Industry	Oil drilling, gas wells, mines (surface or underground), military
Hydrologic modifications	Ditches/channelization, dike/dam/road/railroad bed, water level control structure, excavation, fill, fresh sediment, soil loss/root exposure, wall/riprap, inlets, outlets, pipes (effluent/storm water), impervious surface input (sheet flow)
Habitat modifications	Forest clear cut and selective cut, tree plantation, canopy herbivory, shrub layer browsed, highly grazed grasses, recently burned forest, recently burned grassland, herbicide use, mowing/shrub cutting, trails, soil compaction, off road vehicle damage, soil erosion

The 40-m plot tally (termed “AA-hydro tally” on the field form) recorded any human disturbance activities anywhere within the 0.5-ha AA that were likely to alter hydrology. The entire 140-m plot was too large to census, so 13 square (10 m × 10 m) subplots were systematically laid out to sample this area, which included the AA and a surrounding buffer area. The first subplot was placed in the center of the AA and the remaining 12 subplots were laid out in the buffer along the four cardinal directions (3 in each direction): the first at the edge of the assessment area (40 m from the AA center), the second at the farthest extent of the study buffer (usually 140 m from the center), and the third midway between the other two subplots as depicted in Fig. 2.

### Development of metrics and indices of human disturbance activities and anthropogenic stress

We used the field observations of the presence and proximity of more than 50 pre-determined types of human activity to define two types of indices that quantify human influences on wetlands. The first type of index, the Human Disturbance Activity Index (HDAI), focused on land use and types of human disturbance activities. The second type of index, the Anthropogenic Stress Index (ASI), focused on the influence or stress that these activities were expected to have on wetlands. For the HDAI, we grouped observations of the 50+ human activities into five types (classes) and summed them within and across classes to define five metrics and an overall HDAI. We calculated six ASIs by summing human disturbance activity observations into stressor categories according to their expected effect on each of the six aspects of wetland condition. Both the HDAI and ASI scores were computed at two different plot scales. The 40-m Human Activity Disturbance Index (40m-HDAI) and 40-m Anthropogenic Stressor Index (40m-ASI) scores quantified human activities and their likely stress within the AA, whereas the 140m-HDAI and 140m-ASI scores were computed with data from the entire 140-m plot.

The overall Human Activity Disturbance Index (140m-HDAI) was used for an overall evaluation of disturbance. The 40m- and 140m-HDAI were ultimately used for NWCA reference site screening (Herlihy et al. 2019a). The five metrics contributing to the 140m-HDAI were used to describe the type and intensity of five classes of human activity throughout the US. The six 140m-ASIs were used to quantify the type and intensity of anthropogenic stress in wetlands, and along with the four more proximal 40m-ASIs, they were used to define thresholds of high and low anthropogenic stress that were used to estimate the extent of wetland area in the target population exposed to high levels of these six types of anthropogenic stressors presented in the NWCA public report (USEPA 2016b).

#### 140m-HDAI and its metrics

The NWCA overall 140m-HDAI summarizes the general level of human activities in the 140-m plot that includes the AA and an encompassing buffer area. The tallies of more than 50 types of human disturbance activities were grouped into five classes on the field form: agriculture, residential/urban, industry, hydrology modification, and habitat modification (Table 2). We calculated a separate human activity disturbance metric (140m-HDAIm) for each of the five classes of human activity as the proximity-weighted average of the number of types of human activities tallied in each subplot. Proximity weighting was done in a manner analogous to that used by Kaufmann et al. (1999, 2014a) for calculating stream and lakeshore disturbance indices. Disturbance activities in the one subplot in the center of the AA and the inner ring of subplots had a proximity weight of 1, the middle ring subplots had a weight of 0.44, and the outer ring of subplots had a weight of 0.23 (Fig. 2). Metric scores were calculated as the sum of the number of specific disturbance tallies in each plot times their plot proximity weight, summed across all subplots at the site, and then divided by the total number of plots (13 for almost all sites). If there was one disturbance activity at each of the 13 subplots, the proximity-weighted human disturbance activity metric score would be 0.59. The maximum value observed at a site in the NWCA for any of the five 140m-HDAI metrics was 2.2 but it was rare for a site to have any metric values greater than 1.

We calculated the overall 140m-HDAI by summing the metric scores of the five classes of human disturbance activities (Table 2). The 140m-HDAI summarizes in one continuous variable the intensity of human disturbance activity within the 140-m-radius plot at each site sampled, and was useful as a screening criterion for reference site selection (Herlihy et al. 2019a) and as an overall stressor gradient for NWCA data analyses (Herlihy et al. 2019b; Magee et al. 2019).

#### 140m-ASI

The field categorizations of human disturbance activities were useful for crews filling out forms in the field and describing the types of human activities in and near wetlands, but they are not optimal for assessing stress on wetlands. Because one of the primary objectives of the NWCA was to evaluate the extent and relative risk of impairment from various types of wetland stressors, we reclassified each of the more than 50 human activity and disturbance types listed on the field forms into six categories according to their likely stress on wetland functions: damming, ditching, hardening, filling/erosion, vegetation removal, and vegetation replacement (Table 3). Each field activity or disturbance observation was assigned to one and only one stressor category based on our best judgment of the most significant type of stress imposed by each of the listed anthropogenic disturbance types. Descriptions of the human activities and disturbances assigned to each of the six stressor categories are listed in Table 3.

**Table 3:** Assignment of human disturbance activities from the 140-m plot and 40-m plot checklists to categories based on their likely stress on six aspects of wetland structure and condition

Stressor category	Description	140-m plot checklist items	40-m plot checklist items
Damming	Any field observation related to impounding or impeding water flow from or within the site	Dike/dam/road/railroad bed, water level control structure, wall/riprap	Dikes, berms, dams, railroad beds, sewer outfall
Ditching	Any field observation related to draining water	Ditches, channelization, inlets/outlets, point source/pipe	Irrigation, water supply, field tiling, standpipe outflow, corrugated pipe, box culvert, outflowing ditches
Hardening	Any field observation related to soil compaction, including activities and infrastructure that primarily result in soil hardening	Gravel road, two-lane road, four-lane road, parking lot/pavement, trails, soil compaction, off-road vehicle damage, confined animal feeding, dairy, suburban residential, urban/multifamily, rural residential, impervious surface input	Animal trampling, vehicle ruts, roads, concrete, asphalt
Filling/erosion	Any field observation related to soil erosion or deposition	Excavation/dredging, fill/spoil banks, freshly deposited sediment, soil loss/root exposure, soil erosion, irrigation, landfill, dumping, surface mine	Recent sedimentation, excavation/dredging
Vegetation removal	Any field observation related to loss, removal, or damage of wetland vegetation	Gravel pit, oil drilling, gas wells, underground mine, forest clear cut, forest selective cut, tree canopy herbivory, shrub layer browsed, highly grazed grasses, recently burned forest, recently burned grassland, herbicide use, mowing/shrub cutting, pasture/hay, range	None
Vegetation replacement	Any field observation of altered vegetation within the site due to anthropogenic activities	Golf course, lawn/park, row crops, fallow field, nursery, orchard, tree plantation	None

Field observations of the presence of these activities were used to calculate the six 140-m Anthropogenic Stress Indices (140m-ASIs) and four 40m-ASIs, one for each stressor category and scale of observations

The six stressor categories in Table 3 were chosen to represent the dominant stressor types we observed in the NWCA. Vegetation removal incorporated field form items related to loss, removal, or damage of wetland vegetation. Vegetation replacement included all observations of altered vegetation resulting from human activities within the site. Damming incorporated field observations related to impounding or impeding water flow from or within the site whereas ditching incorporated field observations related to draining of water. Hardening included field observations related to soil compaction, including activities and infrastructure that primarily result in soil hardening.

Assigning the field disturbance checklist items to stressor categories was not always simple. Several rules guided the process:

- Removal of vegetation by either domestic animals (e.g., grazing by cattle) or mechanical devices (e.g., mowing) was considered an anthropogenic stress and was assigned to the vegetation removal category.
- A wholesale change from native vegetation to managed vegetation or crops (i.e., by conversion to lawns, agricultural fields, gardens, landscaping, orchards, nursery, row crops, etc.) was classified as vegetation replacement.
- Disturbances leading to an artificial increase in the elevation of the water table, including human-created surface water and evidence of unnatural damming events (e.g., standing dead pines from human-influenced flooding), were classified as damming.
- Any form of channelized water was considered ditching, including ditches, visual evidence of drainage tiling, piping, and channelization. All were placed in the ditching category.
- Dumping of material (e.g., soil, rocks, and large-scale landfills) or water (e.g., wastewater discharge pipes) was assigned to the filling/erosion category.
- Any activity leading to surface hardening or compaction was placed in the hardening category. This included roads, trails, trampling, and animal tracks.
- Any development (i.e., urban or residential) or disturbance thought to cause compaction was assigned to the hardening category. Exposed pipelines were also included in the hardening category due to probable compaction and hardening (due to pads) during installation, maintenance, and inspections.

For the 140-m-plot tally data (column 3 in Table 3), an Anthropogenic Stress Index (140m-ASI-vegetation removal, 140m-ASI-damming, etc.) was calculated for each of the six stressor categories in the same manner as was done for the five proximity-weighted human activity metrics in the 140m-HDAI. Both the set of five human disturbance activity class metrics contributing to the overall 140m-HDAI (Table 2) and the set of six 140m-ASIs (column 1, Table 3) are sums of the proximity-weighted averages of the number of disturbance tallies within their respective human activity or stressor category.

#### Human Disturbance Activity and Anthropogenic Stress Indices for the 40-m-radius AA plot

We calculated a 40m-HDAI as the simple sum of the number of individual types of human disturbance activities observed anywhere within the 40-m-radius AA. The human disturbance activities targeted in the 40-m AA tally were restricted to things likely to cause changes in the movement or storage of water in wetlands (listed in the fourth column of Table 3), with each of the items being assigned a value of 0.0 (absent) or 1.0 (present). Therefore, the 40m-HDAI does not include tallies related to land uses or habitat modification, and is a general hydrologic alteration index. As 40-m plot data were based on one observation of each disturbance type for the AA as a whole, no proximity weighting was applied as was done for the 140-m plot. Therefore, the summed 40m-HDAI index is an integer (its values are whole numbers). The observed range in the 40m-HDAI across all sites was 0–7 but values above 4 were very rare.

A 40m-ASI was also calculated for each of the four hydrology-related stressor categories (column 4, Table 3) using the human disturbance activity tallies from the 40-m-radius AA. These four 40m-ASIs were calculated in the same manner as the 40m-HDAI, using only the field tally of disturbance activities observed anywhere within the 40-m-radius AA. Specifically, the 40m-ASIs are the simple sums of the number of individual types of human disturbance activities grouped within each of the four categories of hydrology-related stressors (column 4, Table 3).

### Wetland stressor-level thresholds

To make NARS results more understandable to a non-technical audience, we also expressed the continuous numeric ASI scores as classes of low, moderate, or high stressor levels. For purposes of NWCA wetland reporting (e.g., USEPA 2016b) and relative risk analyses (Herlihy et al. 2019c), we combined ASI scores for both the 140-m plot and 40-m-plot (hydrologic stressors only) within each stressor category (Table 3) into a single class score for each stressor category using the threshold definitions in Table 4. For each of the four physical stressor categories (damming, ditching, hardening, and filling/erosion), stressor level was considered low if both the 140-m plot and 40-m plot ASI scores were 0 for that category of stressor. The stressor level within each category was considered high if either the corresponding 140m-ASI was ≥ 0.1 or the 40m-ASI was ≥ 1 for that category. Sites that were not classified as low or high stressor level were considered moderate. For the two vegetation stressor categories (vegetation removal and vegetation replacement), stressor-level thresholds were based only on the 140-m plot scores, as these stressor categories were not included in the hydrologic human disturbance activity tallies done only in the 40-m plot. In summary, a stressor was classified as low at a site only if there were no field observations of human disturbance activities identified within the 40-m plot or the associated 140-m plot; we maintained that any stress on wetlands may be significant.

**Table 4:** Thresholds used to define low, moderate, and high stressor classes from six anthropogenic stress indices in the 140-m plot (140m-ASIs) and four in the AA plot (40m-ASIs)

Stressor indices (40 m-ASI and 140 m-ASI)	Low stressor-level threshold	High stressor-level threshold
Damming, ditching, hardening, filling/erosion	140m-ASI = 0 AND 40m-ASI = 0	140m-ASI ≥ 0.1 OR 40m-ASI ≧ 1
Vegetation replacement, vegetation removal	140m-ASI = 0	140m-ASI ≥ 0.1

Vegetation removal and replacement were not measured in the 40-m-radius AA census tallies, so no AA index scores could be used to define thresholds for those attributes. Values falling between the two thresholds were categorized as “moderate”

### Data analysis

Temporal and measurement variations in the data were assessed using repeat-visit data from a random subset of ~ 10% of the probability sites (*n* = 106) that were visited a second time during the NWCA index period. On average, second visits were made 38 days after the first visit (SD = 23 days). Using the revisit data, we calculated a pooled standard deviation and a signal to noise variance ratio (S:N) for the six 140m-ASIs, the five 140m-HDAI metrics, and the overall 140m-HDAI. S:N is simply the ratio of the variance among all sites (the signal) to the variance within sites from the revisits (the noise) during the same sample year and index period calculated by random effects analysis of variance as described by Kaufmann et al. (1999).

We calculated Spearman rank-order correlations among the six 140m-ASIs to evaluate the strength of their association. We chose Spearman rank-order correlations rather than Pearson’s correlations because we do not assume normality of the data, and Spearman’s coefficient is generally more robust to large data outliers.

The NWCA wetland survey design uses a probability sample based on a wetland area frame, and each site has a sample weight inversely proportional to its probability of being selected. Thus, by using the sample weights in analysis, inference can be made to the entire NWCA target wetland area. In our analyses, all population statistics are weighted so they reflect population areal extent, medians, and percentiles, not simply the unweighted sample statistics. Non-probability sites have a weight of 0 and were not used in these weighted analyses. All data analyses were weighted except for the correlations among stressor indices and the precision estimates (S:N and pooled SD).

## Results

### Index precision

Measures of precision allow researchers to quantify the reproducibility of indices and to evaluate their adequacy for intended applications and interpretations. Our intent was to quantify typical repeat measurement variability for the disturbance activity (i.e., the 140m-HDAIm’s and 140m-HDAI) and wetland stressor (i.e., the 140m-ASIs) indices used in the NWCA. Thus, our estimates of precision and noise variance intentionally include the effects of within-season habitat variation, variation in measurements by and among crews, differences resulting from independent relocation of field plots between visits, and any other kind of field measurement variation resulting from national application of the survey field protocols. At S:N = 0, all the variance observed among sites in the survey can be attributed to “noise.” In terms of its effect on making spatial assessments with survey data, Kaufmann et al. (2014b) report that the adverse effects of noise variance are negligible when S:N *>* 10, become minor as S:N decreases to 6, increase to moderate as S:N decreases to 2, and become severely limiting as S:N approaches 0. S:N can be low due to either low signal and/or high noise. The pooled standard deviation of the revisit samples is the square root of the repeat-visit variance. There are no set standards defining what is a noisy (high) or stable (low) standard deviation. It depends on the purpose for which the data are to be used.

The overall range of the140m-HDAI was 0 to 4, and based on site revisits, had a pooled revisit SD of 0.22 and a S:N of 4.6 (Table 5). For the five human activity disturbance metrics contributing to the 140m-HDAI (Table 5), S:N values ranged from 0.24 (industry) to 9.47 (hydrologic modifications) with only the industrial and habitat modification metrics having a S:N below 3. The S:N for the industrial disturbance metric was low despite having very low noise (pooled SD = 0.013) because it had virtually no signal (the industrial disturbance activity metric values were rarely above 0 anywhere in the NWCA). For the six 140m-ASIs, S:N ranged from 0.86 (140m-ASI-hardening) to 6.46 (140m-ASI-filling/erosion) with only the hardening index having a value below 4 (Table 5). S:N is a relative measure of precision whereas pooled SD is an absolute measure. Among all six 140m-ASIs, pooled SDs ranged from 0.01 to 0.18. Examining our precision results in light of discussions by Kaufmann et al. (1999, 2014b), we believe that the precision of our metrics and indices of human disturbance activity and anthropogenic stress is certainly adequate for the regional or national status and trends analyses of the NWCA.

**Table 5:** Precision of the NWCA 140-m plot anthropogenic stress indices (140m-ASIs), human disturbance activity metrics, and overall human disturbance activity index (140m-HDAI) expressed in terms of the pooled standard deviation (SD) of repeat visits, data range (minimum–maximum), and signal to noise variance ratio (S:N)

Name	Pooled SD	Data range	S:N
Anthropogenic Stress Indices (140m-ASIs):			
140m-ASI-damming	0.028	0–0.608	2.60
140m-ASI-ditching	0.027	0–0.600	4.77
140m-ASI-filling/erosion	0.042	0–1.26	6.46
140m-ASI-hardening	0.132	0–1.87	0.86
140m-ASI-vegetation removal	0.121	0–2.17	4.08
140m-ASI-vegetation replacement	0.050	0–0.960	4.17
140-m plot human disturbance activity metrics:			
140m-HDAIm-agriculture	0.064	0–1.77	8.76
140m-HDAIm-residential/urban	0.037	0–0.888	3.55
140m-HDAIm-industrial	0.013	0–0.462	0.241
140m-HDAIm-hydrologic modification	0.050	0–2.03	9.47
140m-HDAIm-habitat modification	0.180	0–2.26	1.69
Overall Human Disturbance Activity Index:			
140m-HDAI	0.215	0–4.03	4.56

### Prevalence of individual types of human disturbance activities

**Fig. 3:**
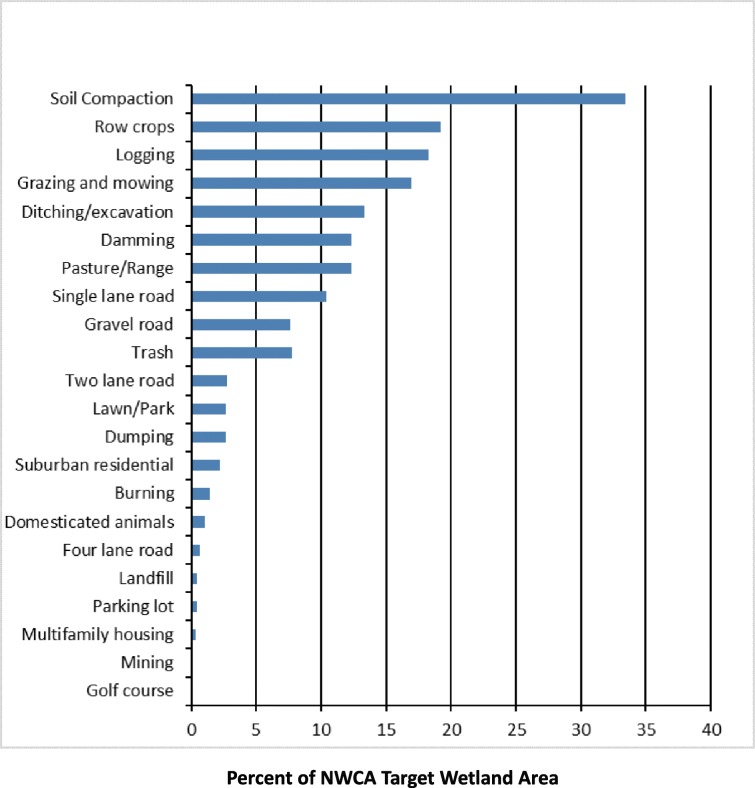
Population estimates of the prevalence of various individual types of human disturbance activities in U.S. wetlands and their buffers, ranked by decreasing percentage of wetland area where these activities are present

Across the conterminous U.S., soil compaction was the most common of more than 50 types of human disturbance activities documented in the field, with more than 30% of the wetland area estimated to have signs of some form of soil compaction in at least one of the 13 plots sampled at each wetland site (Figs. 2 and 3). Seven additional disturbance activities were estimated to be present in 10% or more of the wetland resource area. From most to least prevalent, they are row crop agriculture, logging, domesticated animal grazing and mowing, ditching/excavation, pasture/range, damming, and single-lane roads. Single-lane roads, gravel roads, and two-lane roads were the 8th, 9th, and 11th most common disturbance activities identified, highlighting the influence of roads on wetlands across the US and their potential for fragmenting the landscape in general. Disturbances typical of human habitation (e.g., suburban residential, multifamily housing, golf courses, and parking lots) occurred in only a small proportion of the area of the target population (Fig. 3). A number of disturbance activities were rather rare, being found at less than 1% of wetland area in the nation (e.g., mining and golf courses).

**Fig. 4:**
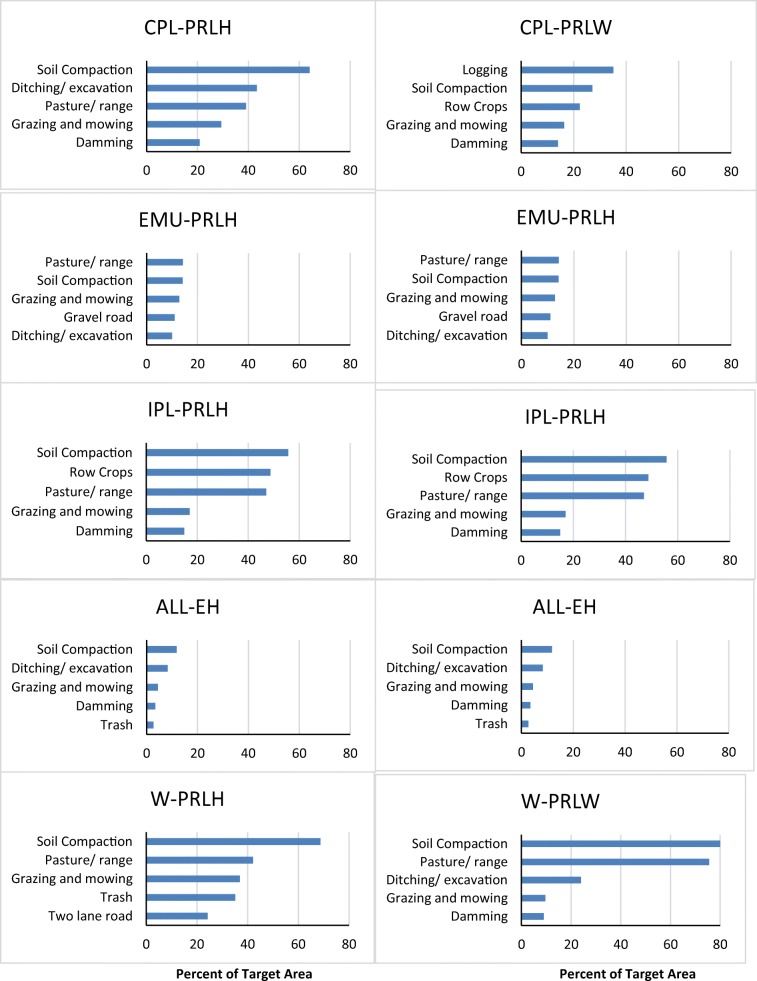
Population estimates of the presence of the five most prevalent of more than 50 individual types of human disturbance activities in each of the 10 NWCA reporting groups, expressed as a percent of wetland area in each group

As with the nation as a whole, soil compaction also topped the list of observed disturbance activities in terms of the percentage of wetland area in six of the 10 reporting groups (Fig. 4). Though compaction and ditching were common in almost all groups, the percentage of wetland area where these types of disturbances were identified varied markedly among reporting groups. In estuarine wetlands, both the herbaceous and woody wetland types were largely free of all of the more than 50 types of disturbance activities we tallied. No single type of disturbance activity was present in more than 16% of the wetland area of the estuarine reporting group as well as in the herbaceous wetlands within the Eastern Mountains and Upper Midwest (EMU-PRLH). By areal extent, the reporting group with the highest level of human disturbance activity was the western woody wetlands (W-PRLW), where both soil compaction and pasture/range were observed in almost 80% of the wetland area (Fig. 4). The groups with the next highest level of human activity were the herbaceous wetland types in the Coastal Plains, Interior Plains, and West where ~ 60% of their wetland area had signs of soil compaction. The second most common human disturbance activities in each of these three reporting groups were ditching/excavation (CPL-PRLH), row crops (IPL-PRLH), and pasture/range (W-PRLH).

### Disturbance within the 40-m plot (AA)

Observations throughout the AA itself were used to calculate the 40m-HDAI, assessing hydrology-related human disturbance activities that were actually within the same area where soil and wetland vegetation condition were assessed. Nationally (data not shown), 64% of the NWCA wetland area had no disturbances, 24% had one disturbance checked, 5% had two disturbances, 4% had three disturbances, and 3% had four or more disturbances (maximum = 7). One or more hydrologic disturbances were noted in 83% of the wetland area in the West, but only 23% of the estuarine wetlands area. The W-PRLH had the highest levels of human activity actually within the AA with a median index value of 2, whereas no other reporting group had a median indicator value for disturbance activities in the AA greater than 0 (data not shown).

### General categories of human disturbance activity

The overall 140m-HDAI and results for its five component metrics summarize the extent of general categories of human activities in and adjacent to US wetlands. Area-weighted median population values of these human disturbance activity metric scores were 0 nationally for agriculture, residential/urban, industry, and hydrologic modification classes (Table 6). Only the habitat modification metric had a non-zero median (0.035) for the US. Only the Estuarine Herbaceous wetlands (ALL-EH) and the herbaceous wetlands of the Coastal Plains (CP-PRLH) and the Eastern Mountains and Upper Midwest (EMU-PRLH) had habitat modification medians of 0. Other than for the habitat modification metric, median values were 0 for all human disturbance activity metrics in all reporting groups except for agriculture in the herbaceous wetlands in the Interior Plains (IPL-PRLH, 0.249) and woody wetlands of the West (W-PRLW, 0.591). Nationally, the 95th percentiles for the metrics of human disturbance activity in and near wetlands ranged from 0 for industrial to 0.685 for habitat modification. Habitat modification also tended to have the highest or near the highest 95th percentile values in each of the individual reporting groups. Agricultural activities were highest in three of the reporting groups, with 95th percentiles of 1.29 in the CPL-PRLH, 0.642 in the W-PRLW, and 0.626 in the IPL-PRLH (Table 6). Residential/urban activities were highest in the W-PRLH (0.182) and CPL-PRLH (0.139). Industrial activities were quite rare everywhere; the 95th percentile of the industrial activity index was 0 for all reporting groups. Only two sites had industrial index scores above 0.1 (maximum = 0.46). Based on 95th percentiles, human activities modifying hydrology in the 140-m plot were highest in the EMU-PRLW (0.334) and W-PRLH (0.273), IPL-PRLW (0.265), and CPL-PRLW (0.257) (Table 6).

**Table 6:** Human disturbance activity metric (140m-HDAIm) population medians by NWCA reporting group and nationally (ALL). Population 5th and 95th percentiles are shown in parentheses

Reporting group code	Agriculture	Residential/urban	Industry	Hydrologic modification	Habitat modification
ALL-EH	0 (0–0)	0 (0–0)	0 (0–0)	0 (0–0.110)	0 (0–0.154)
ALL-EW	0 (0–0)	0 (0–0.105)	0 (0–0)	0 (0–0.223)	0 (0–0.138)
CPL-PRLH	0.018 (0–1.29)	0 (0–0.139)	0 (0–0)	0 (0–0.205)	0 (0–1.618)
CPL-PRLW	0 (0–0.234)	0 (0–0.125)	0 (0–0)	0 (0–0.257)	0.069 (0–0.861)
EMU-PRLH	0 (0–0.061)	0 (0–0.052)	0 (0–0)	0 (0–0.052)	0 (0–0.591)
EMU-PRLW	0 (0–0.103)	0 (0–0.100)	0 (0–0)	0 (0–0.334)	0.023 (0–0.462)
IPL-PRLH	0.249 (0.052–0.626)	0 (0–0.077)	0 (0–0)	0 (0–0.154)	0.128 (0–0.609)
IPL-PRLW	0 (0–0.146)	0 (0–0.034)	0 (0–0)	0.037 (0–0.265)	0 (0–0.387)
W-PRLH	0 (0–0.591)	0 (0–0.182)	0 (0–0)	0 (0–0.273)	0.077 (0–0.925)
W-PRLW	0.591 (0.067–0.642)	0 (0–0.035)	0 (0–0)	0 (0–0.100)	0.518 (0.182–0.608)
ALL	0 (0–0.0591)	0 (0–0.125)	0 (0–0)	0 (0–0.257)	0.035 (0–0.685)

Reporting group codes are defined in Table 1

**Fig. 5:**
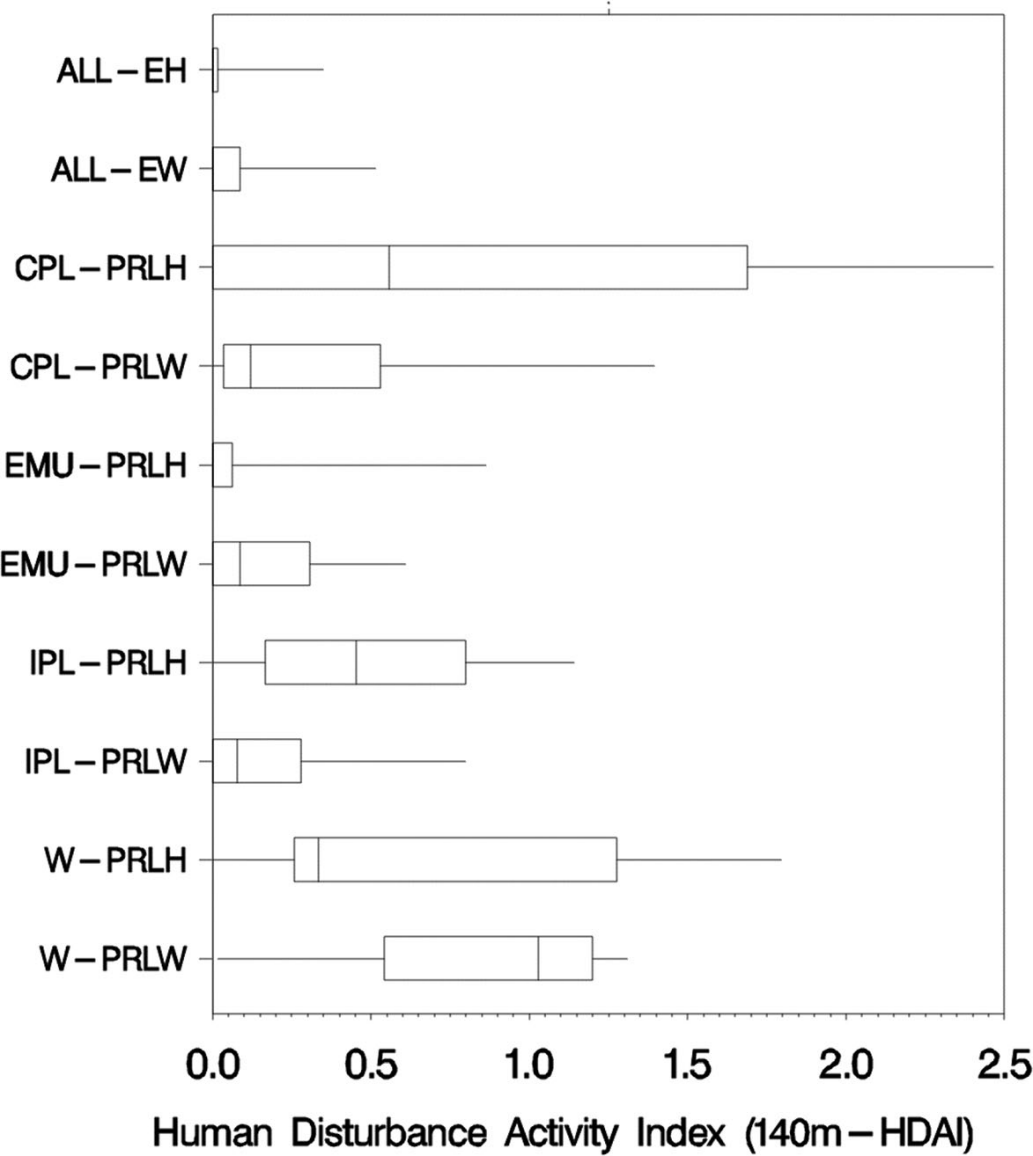
Box and whisker plot of the overall human disturbance activity index (140m-HDAI) by NWCA reporting group. Boxes show the population-weighted 25th and 75th percentiles, the line in the box is the median, and the whiskers show the 5th and 95th percentiles

Among reporting groups, the overall 140m-HDAI, which sums the five-component 140m-HDAI metrics, showed the highest median value in the W-PRLW (1.03) and highest 75th percentile (1.69) and 95th percentile values (2.47) in the CPL-PRLH (Fig. 5). The W-PRLH and IPL-PRLH also showed relatively high levels of overall human disturbance activity. Human disturbance activities were relatively rare in three reporting groups that had median HDAI of 0 (ALL-EH, ALL-EW, and EMU-PRLH). Herbaceous wetlands in the CPL-PRLH had the widest range in 140m-HDAI scores.

### Anthropogenic Stress Indices

Correlations among the six Anthropogenic Stress Indices (140m-ASIs) were mostly very weak (Table 7). Hardening and vegetation removal were the most strongly correlated (*r* = 0.50, *p* < 0.0001). All other relationships were positive but with Spearman correlation coefficients of 0.27 or less. Because of the large sample size (*n* = 1138), all these small correlations were statistically significant (*p* < 0.0001). However, none of the 140m-ASIs other than hardening and vegetation removal shared more than 10% of their variance. Thus, there do not appear to be redundant 140m-ASIs, and the six stressor categories appear to be capturing different aspects of the overall patterns of stressors in wetlands across the US.

**Table 7:** Spearman rank-order correlations (*n* = 1138; *p* < 0.0001 for all) among the six anthropogenic stress indices (140m-ASIs)

	Damming	Ditching	Filling/erosion	Hardening	Vegetation removal	Vegetation replacement
Damming	1	**–**	**–**	**–**	**–**	**–**
Ditching	0.27	1	**–**	**–**	**–**	**–**
Filling/erosion	0.23	0.25	1	**–**	**–**	**–**
Hardening	0.27	0.21	0.26	1	**–**	**–**
Vegetation removal	0.15	0.13	0.24	0.50	1	**–**
Vegetation replacement	0.12	0.12	0.15	0.24	0.24	1

**Fig. 6:**
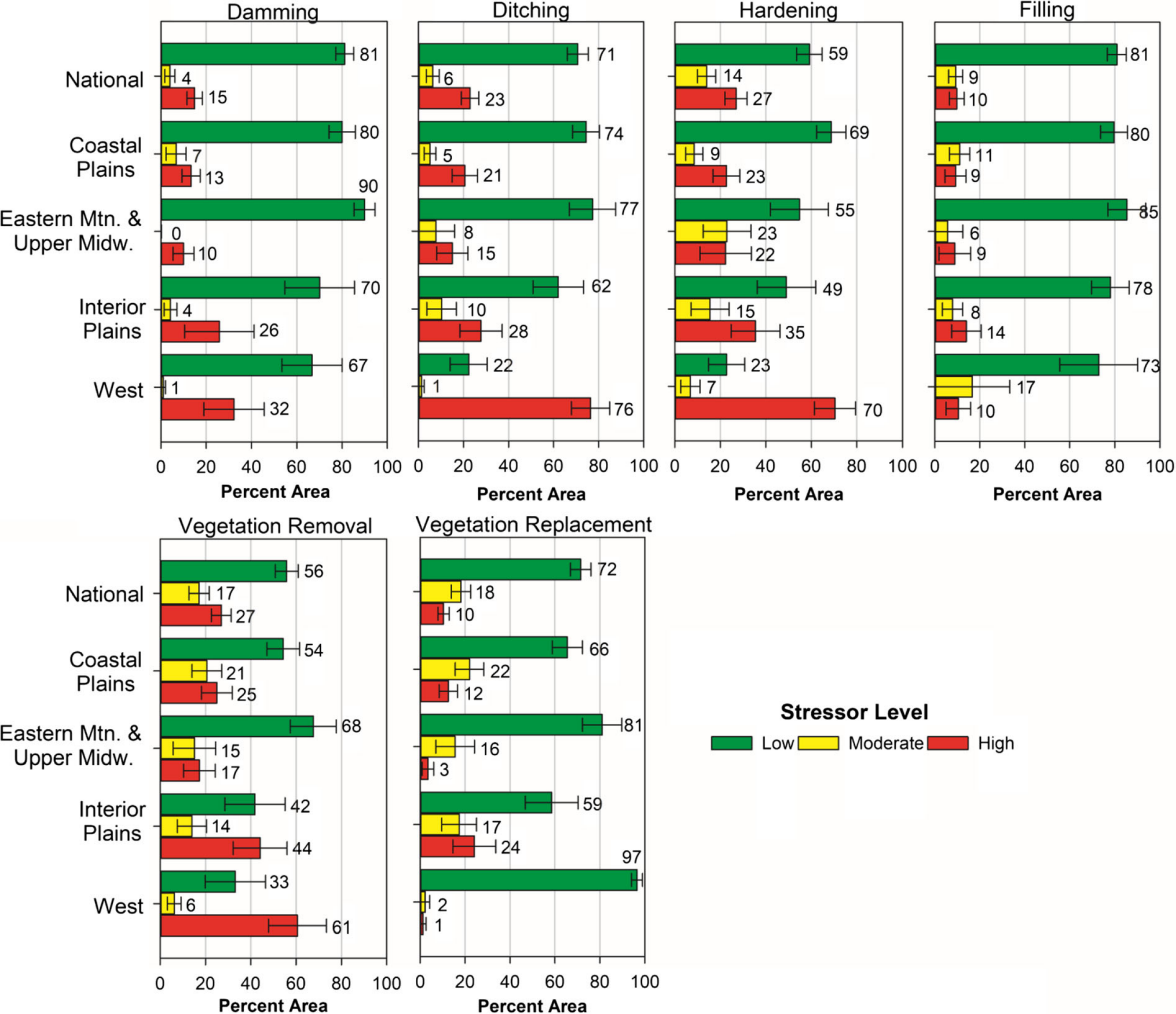
Population estimates of the extent of wetland anthropogenic stress for the six stressor categories, expressed as percentages of the NWCA target wetland area: nationally and by aggregate ecoregion. Low, moderate, and high stressor-level thresholds are defined for the 40-m and 140-m anthropogenic stressor indices (ASIs) in Table 4. Wetland areas and percentage of total are national (251,537 km^2^–100%), Coastal Plains (125,021 km^2^–50%), Eastern Mtn and Upper Midwest (80,762 km^2^–32%), Interior Plains (30,996 km^2^–12%), and West (14,759 km^2^–6%)

The intensity of anthropogenic stress, as measured by the ASIs, across the wetland area and the wetland reporting groups of the US, was expressed in levels of low, moderate, or high stress to make the results more useful to the general public (Table 4). The population estimates of the areal extent and percent of the total wetland area subject to each of the stressor levels are shown in Fig. 6 as they were presented in the NWCA final report (USEPA 2016b). Most of the wetland area in the conterminous US was subject to low anthropogenic stress from any of the six stressor categories we considered (Fig. 6). Vegetation removal and hardening were the stressors with the highest percentage (27%) of wetland area in the high stressor-level class, followed by ditching (23%) and damming (15%).

Stressor-level class estimates varied widely by reporting group (region/wetland type) and type of stressor (Table 8). Both herbaceous and woody groups in the West had high levels of ditching and hardening in over 70% of their wetland area. High levels for all six stressors were much less widespread (< 20% of area) in the ALL-EH, ALL-EW, and EMU-PRLH. The percentage of wetland area with extensive ditching ranged from only 7.2% in the IPL-PRLW to 78% in W-PRLH. Similarly, the percentage of wetland area with substantial vegetation removal ranged from < 5% in the ALL-EH and ALL-EW to over 50% in the CPL-PRLH, IPL-PRLH, and W-PRLW.

**Table 8:** Estimated percent of wetland area in the high stressor-level class by six types of anthropogenic stress, by reporting group, and combined for the nation (ALL)

Reporting group code	Total wetland resource (km^2^)	Damming (%)	Ditching (%)	Filling/erosion (%)	Hardening (%)	Vegetation removal (%)	Vegetation replacement (%)
ALL-EH	20,186	10.2	17.6	4.7	11.1	2.3	0
ALL-EW	2015	0.2	18.3	6.7	13.3	0.3	0.2
CPL-PRLH	15,178	28.1	52.1	23.1	57.5	60.6	14.2
CPL-PRLW	88,464	12.0	16.3	8.3	19.5	24.4	15.2
EMU-PRLH	15,225	12.0	14.3	2.8	11.8	17.9	3.8
EMU-PRLW	65,421	9.5	15.0	10.3	24.7	17.2	3.4
IPL-PRLH	18,611	18.1	41.3	18.7	56.3	54.8	29.9
IPL-PRLW	12,385	37.2	7.2	7.1	4.2	28.1	15.5
W-PRLH	6023	60.8	78.2	16.9	70.1	47.2	0.2
W-PRLW	8037	9.8	75.2	2.2	75.0	75.9	2.1
ALL	251,546	14.9	22.9	9.8	26.9	27.0	10.4

Anthropogenic stressor-level thresholds for each stressor class were based on both 40m- and 140m-ASIs (Table 4). Wetland reporting group codes are defined in Table 1

## Discussion

We sought to derive a field-based measure of the level of anthropogenic stress on wetlands and their local surroundings (buffer). An ideal measure of stress would identify and quantify those aspects of human activity that adversely affect the ecological condition and function of wetlands. To be practical in a national monitoring and assessment program, it was also necessary that field measurements and observations could be collected during a 1-day field sampling visit. Consequently, we faced the challenge of representing the processes of anthropogenic stress by quantifying what are essentially a subset of structural elements (e.g., visible evidence of human activities, land use, soil disturbance). The five wetland human disturbance activity metrics (Table 6) and their resultant sum (140m-HDAI) shown in Fig. 5) are simply proximity-weighted tallies of human activities, land uses, and overt evidence of activities such as soil disturbance and vegetation clearing. On the other hand, the six anthropogenic stress indices (140m-ASIs) (Tables 7 and 8; Fig. 6) attempt to represent processes of anthropogenic alteration of wetland ecological functions or structure (e.g., damming, ditching, filling/erosion, hardening, vegetation removal, and vegetation replacement). Both types of indices use the same set of field observations, but the ASI groupings are overlaid with interpretations (simple models) of the type and level of stress expected from each tallied human activity, based on scientific literature, experience, or first principles.

Cole (2006, 2016) emphasizes the limitations of indices that represent function by structure and strongly argues for research to validate the models and assumptions that underlie their use in monitoring and assessment. Although associations between disturbance indices and biological measures in synoptic surveys such as the NWCA are not true validations (Cole 2016), these results are valuable in focusing public attention, research, management, and restoration on likely causes of ecological damage that is substantial and widespread. Furthermore, associations revealed in these surveys can be used to guide the design of future studies to illuminate mechanisms to validate the postulated causal relationships between human activities and the function and condition of wetlands.

Numerous published findings from ecological surveys demonstrate the power of disturbance indices in predicting habitat degradation and biotic integrity, attesting to their utility in ecological assessment. In a probability survey of Northeastern US lakes, for example, a proximity-weighted measure of near-shore human disturbance activity very similar to NWCA’s 140m-HDAI was shown to be a strong predictor of breeding bird and littoral fish species richness and assemblage structure (Kaufmann et al. 2014c). Similarly, in a near-shore survey of large Brazilian reservoirs, Martins et al. (2015) found a proximity-weighted disturbance measure to be associated with an increase in alien (non-native) fish and shoreline aquatic macroinvertebrates, though Morais et al. (2017) found no similar association for benthic macroinvertebrates in littoral sediments of the same reservoirs. Rowan et al. (2006) modified the USEPA lake habitat assessment field methodology described by Kaufmann et al. (2014b) for application in European Union Water Framework Directive surveys and assessments. Miler et al. (2015) developed an index of human alteration of lakeshore morphology using data collected using the methods of Rowan et al. (2006) on 51 lakes in seven European countries. Their methodology included a proximity-weighted tally of near-shore human disturbance activities similar to that used in the various USEPA surveys. This lakeshore stress index correlated well with a multimetric index of macroinvertebrate assemblage condition, taxonomic diversity, and the presence of disturbance-sensitive taxa (Miler et al. 2015).

The near-equivalent of NWCA’s 140m-HDAI in streams and rivers is the proximity-weighted riparian disturbance index W1_HALL (Kaufmann et al. 1999), calculated from field tallies of human disturbance activities undertaken using the USEPA Environmental Monitoring & Assessment Program (EMAP) or NARS field methods (Peck et al. 2006; USEPA 2007). W1_HALL was an important predictor of excess fine sediments and bed instability in wadeable streams of New Mexico (Jessup et al. 2014), several agricultural regions of the US (Hughes et al. 2010), and the Pacific Northwest US (Kaufmann et al. 2009). Kaufmann and Hughes (2006) showed bed sediment fining and other disturbance-related habitat variables to be the main predictors of fish assemblage biointegrity in the Pacific Northwest coastal streams. Similarly, many other studies have shown W1_HALL to be among the strongest predictors of benthic diatom assemblage structure in wadeable streams of Colorado (Griffith et al. 2005) and the Mid-Appalachian region (Hill et al. 2001). W1_HALL was also among the strongest predictors of macroinvertebrate assemblage composition and structure in lotic waters of Central California (Griffith et al. 2003; Pan et al. 2006) and Colorado (Griffith et al. 2005), and in wadeable streams of the south-central Brazilian savanna (Ligeiro et al. 2013; Ferreira et al. 2014; Macedo et al. 2016).

Based on published literature reporting numerous and widespread strong associations of biotic assemblage structure and composition with human disturbance measures similar to NWCA’s 140m-HDAI, we feel confident that the wetland HDAIs and ASIs we describe are reasonable indices to represent anthropogenic stress on wetlands in the NWCA.

### Human disturbance activity in wetlands

The distributions of 140m-HDAI values show clear differences among the 10 reporting units, and the median 140m-HDAI values are useful in ranking regions and wetland types by the overall level of human activity and disturbances in wetlands and their buffer areas (Fig. 5). The woody and herbaceous estuarine wetlands and the Eastern Mountain herbaceous wetland groups had the lowest human activity levels, with median 140m-HDAI values of 0. At the other extreme, the highest median 140m-HDAI values (0.5 to 1.0) were in the West-woody, the IPL-herbaceous, and West-herbaceous groups. The highest observed individual wetland 140m-HDAIs were in the CPL-herbaceous (2.5) and the West-herbaceous (1.8) groups.

Overall, we found that urban disturbances were a minor contributor to both extent and magnitude of disturbance to wetlands nationally, as expressed by the overall human disturbance index (140m-HDAI). Much of the wetland areas in the contiguous US, and particularly the estuarine wetlands along the east coast, are part of vast wetland complexes that tend to make human activity and development difficult. The estuarine reporting groups contained very little human activity of any type. Median 140m-HDAI scores were 0 and the 95th percentile did not exceed 0.5 for ALL-EW. Agriculture (pasture, row crop agriculture) is an important land use in the regions containing the estuarine and also the Eastern Mountain and Upper Midwest reporting groups, but the magnitudes of human activity were relatively low and the areal percentage of wetlands and their buffer areas affected by agriculture was low in these reporting groups. Agriculture was also a dominant land use in the CPL-PRLH and the IPL-PRLH reporting groups, but their median 140m-HDAI also ranked them very high in overall human disturbance activities. The highest median 140m-HDAIs were for the W-PRLW group (1.0) in the relatively drier West, where fewer wetlands exist, and for the CP-PRLH group (0.5), the herbaceous wetlands of the densely populated Coastal Plains (Fig. 5). We found both the extent and magnitude of human disturbance activity to be generally greater in the CPL-PRLH, IPL-PRLH, and the two western reporting groups (Figs. 5 and 6). In those groups, top disturbances ranged from 55 to 80% of the wetland areal extent.

### Anthropogenic stress on wetlands

In the US as a whole, the percentages of wetland area with high anthropogenic stress measured by 140m-ASIs were relatively uniform across the six stressor categories, varying from approximately 10% (~ 25,000 km^2^) for both filling/erosion and for vegetation replacement to more than 25% (> 63,000 km^2^) for hardening, vegetation removal, and ditching (Fig. 6). High levels of activities related to surface hardening (e.g., soil compaction, roads) were found in 27% (68,000 km^2^) of wetland area nationally. These activities affect how water flows in and out of wetlands and the amount of water that enters and stays within wetlands, potentially impacting plant productivity, nutrient cycling, and overall physical habitat. Similarly, 27% (68,000 km^2^) of wetland area nationally had high levels of activities related to plant removal. Removal or loss of vegetation, resulting from grazing, mowing, or forest clearing, may increase sediment, nutrient, and pollutant loads entering and remaining in a wetland. Nearly one-quarter (23%; 58,000 km^2^) of wetland area nationally had high levels of ditching (Fig. 6). Ditching affects how water flows in and out of wetlands, potentially impacting plant productivity, nutrient cycling, and physical habitat.

Given the diversity of geomorphology, water regime, and land use in the US, it is not surprising that the dominant types of anthropogenic stress and the percentages of highly stressed wetland area varied widely when assessed by separate reporting units (Table 8). Individual category patterns across reporting groups were similar to those for overall 140m-HDAI (Fig. 5) and its component metrics (Table 6). This concordance is expected, because the stress indicators were derived from the same set of observations on the type and intensity of human activities, and these differ strongly across regions and wetland types. The wetland groups with the lowest stress were the estuarine wetlands (ALL-EH and ALL-EW) and the herbaceous wetlands in the Eastern Highlands (EMU_PRLH), where percentages of wetland area with high stress were less than the national average for all six stressors (Table 8). By contrast, the most generally stressed wetlands were the herbaceous wetlands in the Coastal and Interior Plains (CPL_PRLH and IPL-PRLH), where percentages of wetland area with high stress were greater than the national average for all six stressors.

### Comparison with lake and stream assessments

The patterns in human disturbance activities assessed in the field by NWCA in the wetland 140-m plots were similar to those measured using other proximity-weighted tallies of human disturbance activities in the near lakeshore zone by the National Lakes Assessment (NLA, Kaufmann et al. 2014a) and the near-stream/riverbank zones by the National Rivers and Streams Assessment (NRSA, USEPA 2016c). For the conterminous US as a whole, the range in areal percentage of wetlands with high anthropogenic stress within the 140-m plot for the six categories (9.8–27%) encompassed that observed for the number of lakes (17%) and stream/river length (11%) with high levels of near-shore or near-bank disturbance. On the other end of the disturbance gradient, however, the percentage of wetland area with low disturbance (56–81%) was considerably higher than that for the number of lakes (34%) and stream/river length (39%) in the low disturbance category.

All three assessments showed the Upper Midwest and Eastern Highlands to be among the regions with the lowest disturbance. In the NLA and NRSA, only 9% of lakes or stream miles in these regions had high disturbance. Low percentages of highly disturbed lakes and streams characterized the Northern Appalachians (11–14%), while disturbances were greater in the Southern Appalachians, where 24% of lakes and 26% of stream miles had high levels of disturbances. Like the NWCA, the other two assessments showed high disturbance in the Interior Plains (Central Plains) ecoregions. For both lakes and streams, the percentages of highly disturbed waters were greatest in the Northern Plains (35% of lakes, 61% of stream miles) and the Southern Plains (30% of both lakes and stream miles). The NWCA showed a large percentage of highly disturbed wetland area in the Western US (75–78% with ditching, damming, or vegetation removal), whereas the lake and stream assessments reported high percentages of disturbed lakeshores and stream banks in the Xeric ecoregions of the West (32% and 46%, respectively), but low to moderate percentages of waters with high disturbance in the Western Mountains (12% of lakes, 18% of stream miles).

### Utility and relevance of the NWCA disturbance and stress indicators

The percentages of wetlands with high levels of anthropogenic stress express the relative extent of these stressors, summarizing how widespread or common each stressor is. Specifically, the NWCA population estimates of relative extent quantify the amount of wetland area in the US having high, moderate, or low levels of each category of wetland stressor, based on the combined 40m- and 140m-ASIs. A stressor with a high relative extent (i.e., a large area of the wetland resource with high stress) in the US suggests cause for national concern. The NWCA relative extent findings showed that among all the physical, chemical, and biological stressors examined, vegetation removal, surface hardening, and ditching were the most pervasive stressors across the nation. High levels of vegetation removal and surface hardening were found for 27% of the wetland area, while 23% of wetland area had high levels of ditching. Herlihy et al. (2019c) estimated the relative and attributable risk of stressors in the NWCA by examining the strength of association between biological condition, quantified by the Vegetation Multimetric Index (VMMI, Magee et al. 2019), and the various stressors quantified by the NWCA. They reported that wetland sites with high ASI scores from vegetation removal and surface hardening were about twice as likely to have poor biological condition as those with low or moderate levels. At the national level, ASI scores denoting high levels of vegetation removal, surface hardening (e.g., soil compaction), ditching, damming, filling/erosion, and vegetation replacement were also more likely to have lower VMMI scores than those not subject to those stresses. The national extent of wetland stressors and their association with poor biological condition support initiating or continuing national efforts to reduce or mitigate the effects of vegetation removal, surface hardening, and ditching. Although some stressors, such as filling/erosion, might not be as widespread nationally, they can be dominant in smaller regions, suggesting a need for localized assessments and management actions to target stressors that are locally important.

### Insights from other studies

Results from regional studies show mixed results regarding the strength of associations between wetland biota and human activities adjacent to wetlands and those at a larger landscape scale. Based on a study of 55 wetlands in the US Interior Plains state of Oklahoma, for example, Bried et al. (2016) reported that the site-scale buffer disturbance evaluated by the NWCA disturbance index (140m-HDAI) was not a good predictor of the condition of wetland vegetation. In some regions, however, floral or faunal assemblages may be more strongly influenced by the type and proximity of overt human activities assessed by the NWCA disturbance measures. In Ohio, by contrast, metrics of habitat alteration, soil disturbance, and wetland human development (Ohio Rapid Assessment Method) in a site-scale buffer area similar to that used by NWCA were consistently strong predictors of wetland vegetation condition (Stapanian et al. 2013) and amphibian biotic integrity (Micacchion et al. 2015) in shrub and forested wetlands. Their findings emphasized greater influence of site-scale disturbance compared with broader landscape measures based on remote imagery and contrasted with an earlier Ohio wetland study by Mack (2006) showing negative correlations with broader landscape disturbance measures. Miller et al. (2016) assessed wetland condition in the northeastern US, using NWCA methods, and reported that a multimetric index of wetland vegetation condition was best predicted using both landscape and wetland site-scale buffer measures of disturbance. Despite the regional variation in the strength of association of wetland biota with proximal and more remote human activities reported in regional studies, we agree with the general conclusion of Bried et al. (2016) that the use of anthropogenic stress metrics should complement, rather than replace, direct bioassessments such as the NWCA vegetation condition analysis described by Magee et al. (2019).

These varying results concerning the predictive power of various site-scale versus landscape-scale wetland disturbances are not surprising, as influences on wetland condition differ by region, wetland type, and the general level and pattern of disturbance over the broader landscape (Cunningham and Johnson 2016), as well as the history of past human activity and natural disturbances (Schweiger et al. 2016). Moreover, the overt presence of human activity in wetlands or their surrounding landscape does not provide a complete assessment of the biological, chemical, climatic, and land use stress on wetlands. In a particular year or region, current NWCA and other wetland vegetation condition indicators may be influenced by natural interannual variation in water status (drought, floods) that may mask or magnify the effects of anthropogenic stress on wetlands and their buffer areas. Furthermore, wetland vegetation composition, structure, and integrity may change in response to both natural and anthropogenic changes in precipitation and runoff. Our measures of the type and intensity of human activities and stress in wetland buffer areas can help to disentangle the human- and non-human-associated changes in wetland flora and fauna (Schweiger et al. 2016).

Individual wetlands commonly occur in large complexes or they can occupy large areas; as a result, the NWCA’s 0.5-ha assessment areas may be completely surrounded by additional wetland area and buffered from disturbance. With saturated soils and standing surface waters, wetlands are not suitable for urban development or many types of agriculture unless they are drained or filled. We emphasize that wetlands that have already been filled, flooded by reservoirs, drained, or otherwise converted to human uses (e.g., cropland, cities) do not appear in wetland inventories. Consequently, these lost wetlands and the causes of their loss are not included in the NWCA. By the mid-1980s, only 103 million acres of wetland remained out of an estimated 220 million acres in the conterminous US during the 1600s (Dahl et al. 1991). Agriculture (including crop production and domesticated animal grazing opportunities) was the primary motivation for reclaiming the land (removing wetlands), but urban development and impoundment or dredging of waterways for navigation further decreased wetland acreage (Dahl and Allord 1996; Holland et al. 1995). Of the wetland area remaining in the US and assessed by this study, much is subject to the same types of disturbance and stress. A challenge for the NWCA is to incorporate the loss (or gain) in wetland resource area into its national and regional assessments of wetland condition. This challenge might be addressed without modifying the NWCA design, but by coordinating the interpretation of survey results with the USFWS S&T program, which reports on gains and losses of wetland area.

## Conclusions

We derived five proximity-weighted metrics and an overall index (HDAI) to quantify the intensity of human disturbance activities on wetlands and their surrounding areas, employing field tallies of over 50 individual types of human activity on 1138 wetland sites, of which 967 were probability sites representing 251,546 km^2^ of wetland area in the 48 contiguous states of the US. Based on published knowledge of the likely influence of each type of human activity, we then developed a group of indices to quantify the levels of anthropogenic stress on wetlands. Estimates of precision based on repeat-visit data indicated that these metrics and indices are precise enough for regional and national assessments. We classified the levels of anthropogenic stress (high, moderate, low) at each NWCA sample site based on nationally consistent rules informed by literature, management, and first principles. These stressor determinations were used to make population estimates of the levels of anthropogenic stress on wetlands throughout the conterminous US and by ecoregions and wetland types. Among the six stressor categories assessed nationally, the percentages of wetland area having high levels of disturbance ranged from 10% associated with filling/erosional activities to 27% from vegetation removal activities. The proportion of wetland area with no signs of disturbance (140m-HDAI = 0) was 29% for the nation, but varied widely among the different wetland ecoregions/types we assessed. Estuarine wetlands were mostly undisturbed (70% of area). On the other hand, among non-estuarine wetlands, only 8% of the wetland area in the West, 15% of the Interior Plains, 22% of the Coastal Plains, and 36% of the Eastern Mountains and Upper Midwest were undisturbed. Woody wetlands in the West were the most heavily disturbed reporting group, with over 75% of their area subjected to high levels of ditching, hardening, and vegetation removal. The NWCA offers a unique opportunity to quantify the type, intensity, and extent of human disturbance activities in and around wetlands, and to assess their likely stress on the physical and biological integrity of wetlands at continental scales.
